# Low-dose radiation treatment for painful plantar enthesophyte: a highly effective therapy with little side effects

**DOI:** 10.1186/s40001-022-00642-x

**Published:** 2022-02-23

**Authors:** Freddy Djiepmo, Bálint Tamaskovics, Edwin Bölke, Matthias Peiper, Jan Haussmann, Judith Neuwahl, Danny Jazmati, Kitti Maas, Livia Schmidt, Roman Gelzhäuser, Christoph Schleich, Stefanie Corradini, Klaus Orth, Martijn van Griensven, Amir Rezazadeh, Kimia Karimi, Wilfried Budach, Christiane Matuschek

**Affiliations:** 1 Department of Radiation Oncology, Heinrich Heine University, Dusseldorf, Germany; 2 Medical Faculty, Heinrich-Heine-University, Dusseldorf, Germany; 3 Institute of Mathematical Statistics and Probability Theory, Heinrich-Heine-University, Dusseldorf, Germany; 4 Radiology Dusseldorf/Viersen, Ernst-Schneider-Platz 1, Dusseldorf, Germany; 5 Department of Radiation Oncology, University Hospital, LMU Munich, Munich, Germany; 6 University of Hannover, Hannover, Germany; 7 Department cBITE, MERLN Institute for Technology-Inspired Regenerative Medicine, Maastricht University, Maastricht, The Netherlands; 8 Klinik Für Strahlentherapie Und Radiologische Onkologie, Heinrich-Heine-Universität, Moorenstr. 5, 40225 Düsseldorf, Germany

**Keywords:** Plantar enthesophyte, Heel spur, Radiation therapy, Benign disease, Pain, Photon therapy, Electrons

## Abstract

**Aim:**

Plantar enthesophyte is a common degenerative disorder. Surgical and medical treatment options are associated with either poor outcome or high percentage of relapse. Observations have indicated a beneficial effect of radiation therapy. We therefore wanted to evaluate pain reduction using orthovolt or cobalt-based radiation treatment for painful plantar enthesophyte and determine long-term response as well as prognostic parameters in this condition.

**Methods:**

We identified a total of 102 consecutive patients treated for a total of 117 symptomatic heel spurs. 59 patients were treated with cobalt radiation, 31 patients with orthovolt therapy and 12 patients with both radiation systems. Primary outcome measure was pain reduction being scored using the modified Rowe Score prior therapy, at the end of each treatment series as well as after 6 weeks. Secondary outcome measure was long-term outcome, evaluated in patients with a follow-up period of longer than 3 years.

**Results:**

Before radiation therapy, 61 patients (60.4%) had a score of 0, significant strong pain. At the time of completion of radiation treatment, 3 patients (2.7%) were pain-free (score of 30), whereas 8 patients (7.9%) had still severe pain (score 0). 6 weeks after radiation therapy, 33 patients (32.7%) were pain-free and 8 patients (7.9%) had severe pain (score 0), while at the time data of collection, 74 patients (73%) were free of pain and 1 patient (1%) had strong pain (score 0). Duration of pain before the start of radiation treatment was a significant prognostic factor (*p* = 0.012) for response to treatment.

**Conclusion:**

Radiotherapy of painful plantar enthesophyte is a highly effective therapy with little side effects providing long-term therapeutic response. The only significant prognostic parameter for response to treatment is the duration of pre-radiation therapy pain. Early integration of radiation therapy in the treatment seems to result in superior pain reduction.

## Introduction

**Fig. 1:**
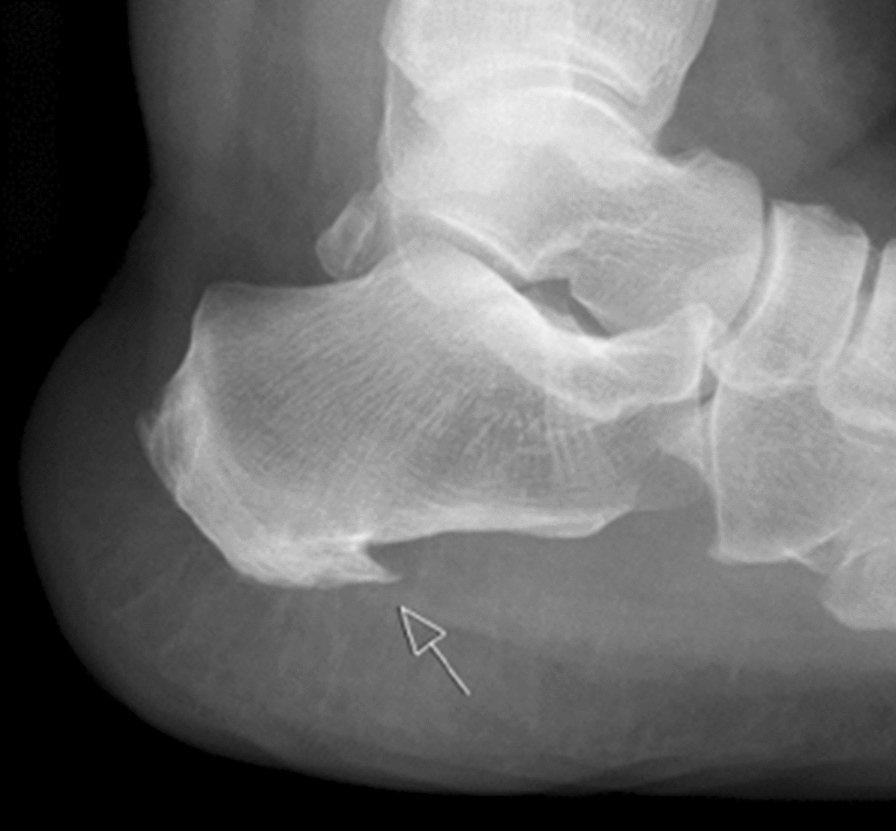
X-ray of plantar enthesophyte. The X-ray of the calcaneus shows a heel spur with an inflammatory reaction surrounding the insertion of the inferior aponeurosis (arrow)

Plantar enthesophyte is a painful degenerative disorder evolving over a long time [1–4] and is localized at the tuber calcanei [5] (Fig. 1).

Plantar enthesophyte, often described as heel spur, was first described by Plettner [6]. He investigated radiological findings of exostosis at the plantar sole and parts of the calcaneus. Nowadays, plantar enthesophyte are one of the most common causes of pain in the heel region.

The plantar enthesophyte develops as a reactive formation of bone due to mechanical stress with degenerative changes and microtraumas at the insertion of the tendons of the abductor hallucis and the brevis flexor digitorum muscles as well as the plantar aponeurosis and can therefore be identified as a result of a plantar fasciitis. Nevertheless, this term is misnomer, since the plantar fascia is an aponeurotic rather than a fascial layer. Plantar enthesophytes are located at the calcaneus where the plantar aponeurosis inserts at the medial tuber calcanei or at the insertion of the Achilles tendon in a dorsal position. Combinations of the two locations have also been described [7]. Risk factors are female sex (female:male = 3:1) [8], age above 40 years, obesity, weight bearing occupation [9] as well as anatomical deformity of the foot such as talipes valgus, talipes planus and flatfoot [7].

The majority of patients with plantar enthesophyte remain asymptomatic. If clinically apparent, typical symptoms are pressure pain at the medial part of the arch of the foot radiating to the calf and the sole of the foot. The pain is often described as burning or stinging, emerging while walking barefoot or after resting. Therefore, the diagnosis of plantar fasciitis is mostly a clinical one. Any further diagnostic approach should be tailored according to the clinical picture and includes foremost plain X-ray. Additional diagnostical regimen may include technetium bone scintigraphy (revealing a fasciitis) as well as MRI, though both techniques are rarely used.

If symptoms are present, physical activity may improve them. Conservative or invasive therapeutic options have been implemented in the therapy of plantar enthesophytes. Primary treatment usually consists of conservative management including orthotics, local or systemic analgesics, corticosteroids [10], antiphlogistics [11, 12], physiotherapy [13, 14] and stretching exercises [14, 15]. Massages, local thermotherapy, ultrasound and electrical stimulation therapy are often prescribed for several times [16, 17]. Nafe and other authors were able to demonstrate a temporary pain reduction effect of extracorporeal shockwave therapy [3, 4, 18–22]. However, at least in Germany, the latter therapeutical approach is rarely compensated by health insurances. Despite high rates of recurrences, surgical management was used for chronic pain condition after conservative therapy, but is usually not a standard method in the treatment of a painful plantar enthesophyte [1, 10, 23, 24]. Despite an increasing number of physicians successfully using radiation treatment in degenerative inflammatory disorders, the role of radiotherapy in this context is currently under discussion [25–27].

In this retrospective study, we tried to clarify the following questions:

Which parameters influence the clinical outcome (pain reduction) in the radiation treatment (RT) of plantar enthesophytes? We identified radiation parameters (single fraction and overall fraction dose, overall treatment time, field size (radiologically vs. clinically guided fields), type of radiation) as well as clinical parameters (sex, age, treatment prior to radiation, duration of pain prior to radiation).

## Patients and methods

During 2 years, 102 patients were treated for overall 117 plantar enthesophytes. 28 male (27.2%) und 74 female (71.8%) patients with a mean age of 51.5 years (range: 20–80 years, median 52 years) were included in this study. 87 patients had a uni-lateral plantar enthesophytes (55 right, 32 left) and 15 patients had bilateral plantar enthesophytes. The correct diagnoses were confirmed using X-ray by two radiologists. 59 patients were treated with cobalt radiation (^60^Co) and 31 with X-rays with an orthovoltage irradiation (300 kV). 12 patients were treated with both energies.

Radiation treatment was indicated in patients aged 20 to 50 years when conservative methods failed to reduce clinically evident pain and patients experienced a loss of function in everyday life.

Radiation treatment with both techniques consisted of 6 fractions of 0.5–1 Gray (Gy) given 2–3 times a week up to total dose of 3 or 6 Gy. In detail, patients being treated with cobalt radiation were irradiated 3 times a week with a dose/fraction of 0.5 or 1 Gy up to a total dose of 3 or 6 Gy, depending on their pain intensity. Higher doses of radiation were prescribed for patients with persistent and severe pain. They always received a total of 6 fractions. Orthovoltage irradiation was applied 2 to 3 times per week with a dose/fraction of 1 Gy and a total dose of 3 or 6 Gy, resulting in a total of 3 or 6 fractions which were applied. In addition, the therapeutic effect of local steroid injection was evaluated. In 12 patients both 60-cobalt and orthovoltage therapy were administered. One reason for it was the availability of the machine. 12 patients started with orthovoltage irradiation, but as the machine was unavailable cobalt irradiation (60Co) was prescribed.

If only temporary or partial pain relief was obtained, a second course was offered to the patients. This was given after a median time gap of 5.4 (range 3–12) months after initial treatment.

Pain and function were evaluated using a modified score of Rowe et al. [28]. Table 1 shows the criteria used in this measurement. Patients were contacted directly or interviewed by telephone and completed the questionnaire retrospectively for the following four points in time: (1) before start of radiation; (2) on the last day of radiation; (3) 6 weeks after radiation; (4) during follow-up (at least 3 years after radiation treatment).

**Table 1:** Modified pain and function score according to Rowe et al. [32]

	Criteria	Response level	Score
Pain	At rest	None	30
		Mild	20
		Moderate	10
		Severe	0
	Pain in motion	None	0
		Mild	30
		Moderate	20
		Severe	10
	Pain when applying pressure to the heel	None	0
		Mild	30
		Moderate	20
		Severe	10
Medical aids		None	15
		Orthopedic insoles, sole padding	10
		One walking aid (cane or forearm support)	5
		Two walking aids	0
Everyday activities		Normal, no constraints	15
		Small constraints	10
		Moderate constraints	5
		Complete constraints	0
Gait		No limping, normal gait with no constraints	20
		Mild pain and limping after > 1 km	10
		Moderate pain and limping after < 1 km	5
		Severe pain, no normal gait possible	0

Score 120–140: excellent; Score 90–120: good; Score 60–90: moderate; Score 30–60 mild; Score 0–30 severe

We statistically evaluated radiation-associated factors (single and overall dose, treatment time, field size, type of radiation, pain medication) and clinical parameters (sex, age, prior treatment before radiation, duration of pain before radiation treatment) possibly influencing the clinical outcome using Microsoft-Excel 2010 and SPSS-20.

Data were expressed at mean ± standard error of the mean (SEM). Statistical significance was assessed by Mann–Whitney *U* and *t*-test. *p*-values less than or equal to 0.05 were considered statistically significant (*p ≤ 0.05, **p ≤ 0.01, ***p ≤ 0.001). With the Kruskal–Wallis test, we examined the influence of radiotherapy on pain relief in different groups. Normal distribution was assessed using the D’Agostino–Pearson and Kolmogorov–Smirnov test.

## Results

In this retrospective analyses, we identified 102 consecutive patients treated in our institutions within 2 years. Table 2 shows our patients characteristics. Diagnosis was performed using established clinical parameters. Ideally, confirmation of diagnosis with MR technique revealing marrow and soft tissue edema along the proximal plantar fascia may underline this. Since clinical evidence and treatment decision is routinely done without this diagnostic feature because it is not necessary for clinical treatment, it is not performed widely. 102 patients were treated for a number of 117 symptomatic plantar enthesophytes. 59 patients were treated with 60-cobalt therapy, 31 patients with orthovolt therapy and 12 patients with both radiation systems.

**Table 2:** Patients characteristics

Criteria	No. of patients	(%)
Patients	102	100
Female	74	71.8
Male	28	27.2
Spurs	117	100
Right-sided	55	54.1
Left-sided	32	31.6
Bilateral	15	14.3
Pre-treatment	134	100
Orthopedic insoles	48	35.8
Corticoid infiltrations	25	18.6
Antiphlogistics (oral)	34	25.3
Physiotherapy	3	2.2
Shockwave therapy	6	4.5
Massage	5	3.7
Ultrasound therapy	4	2.9
Antiphlogistics (ointment)	5	3.7
Corticosteroids (oral)	2	1.5
Surgical therapy	2	1.5

**Fig. 2:**
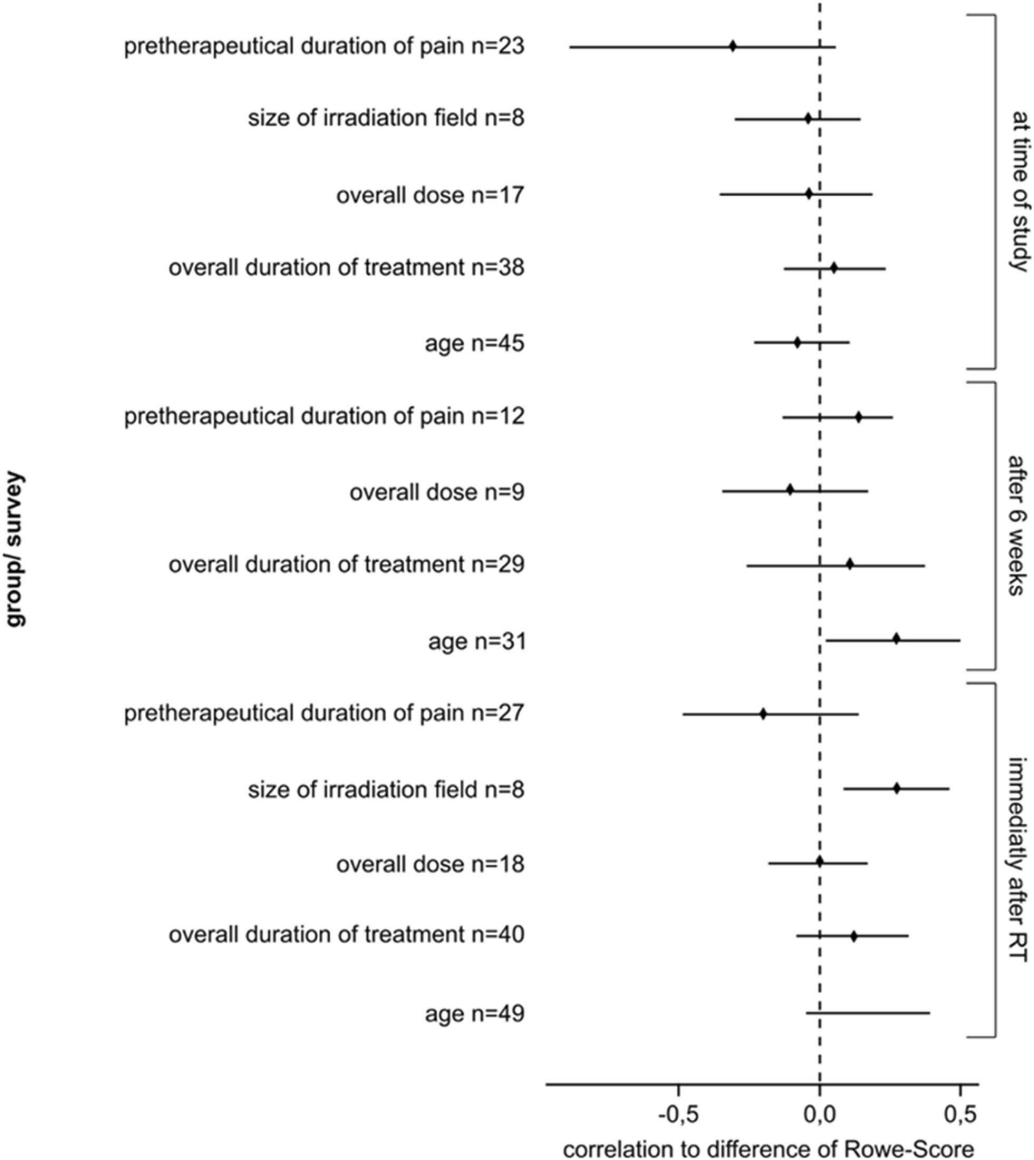
Graphic depiction of the sum score before, after RT and at final data collection

Prior to RT treatment, 100 patients (99%) presented with a Rowe S-Score between 0 and 30 (severe pain) and only 1 patient (1%) had a S-Score between 30 and 60 (mild pain) (Fig. 2).The pretreatment group of 134 patients was set to 100% because it includes patients with double or more different pre-treatments.

On the last day of RT treatment, 21 patients (20.8%) achieved a S-Score between 0 and 30 (mild to no improvement), 26 patients (25.7%) a S-Score between 30 and 60 (mild pain improvement), 24 patients (23.7%) a S-Score between 60 and 90 (mild to moderate improvement) and 29 patients (28.7%) had a S-Score between 90 and 120 (good pain response). Only 1 patient (1%) achieved a complete pain response with a S-Score between 120 and 150.

If patients were contacted directly or interviewed by telephone and completed the questionnaire retrospectively for 6 weeks after radiation, during follow-up (at least 3 years after radiation treatment) 53 patients (52.5%) had a S-Score of 0–30 (mild to no improvement of pain), 2 patients (2%) a S-Score between 30 and 60 (mild improvement of pain), 7 patients (6.9%) a S-Score of 60–90 (mild to moderate improvement of pain) and 6 patients (5.9%) had an S-Score of 90–120 (major improvement of pain). Overall, 33 patients (32.7%) had a complete pain remission with a S-Score of 120–150.

At the time of data collection, the results of a telephone questionnaire were the following: 19 patients (18.8%) reported a S-Score of 0–30, 2 patients (2%) a S-Score between 60 and 90, 7 patients (6.9%) a S-Score of 90–120 and 73 patients (72.3%) had a S-Score of 120–150. No patient reported a S-Score of 60–40 and 14 patients (13.9%) experienced a S-Score of 0.

**Fig. 3:**
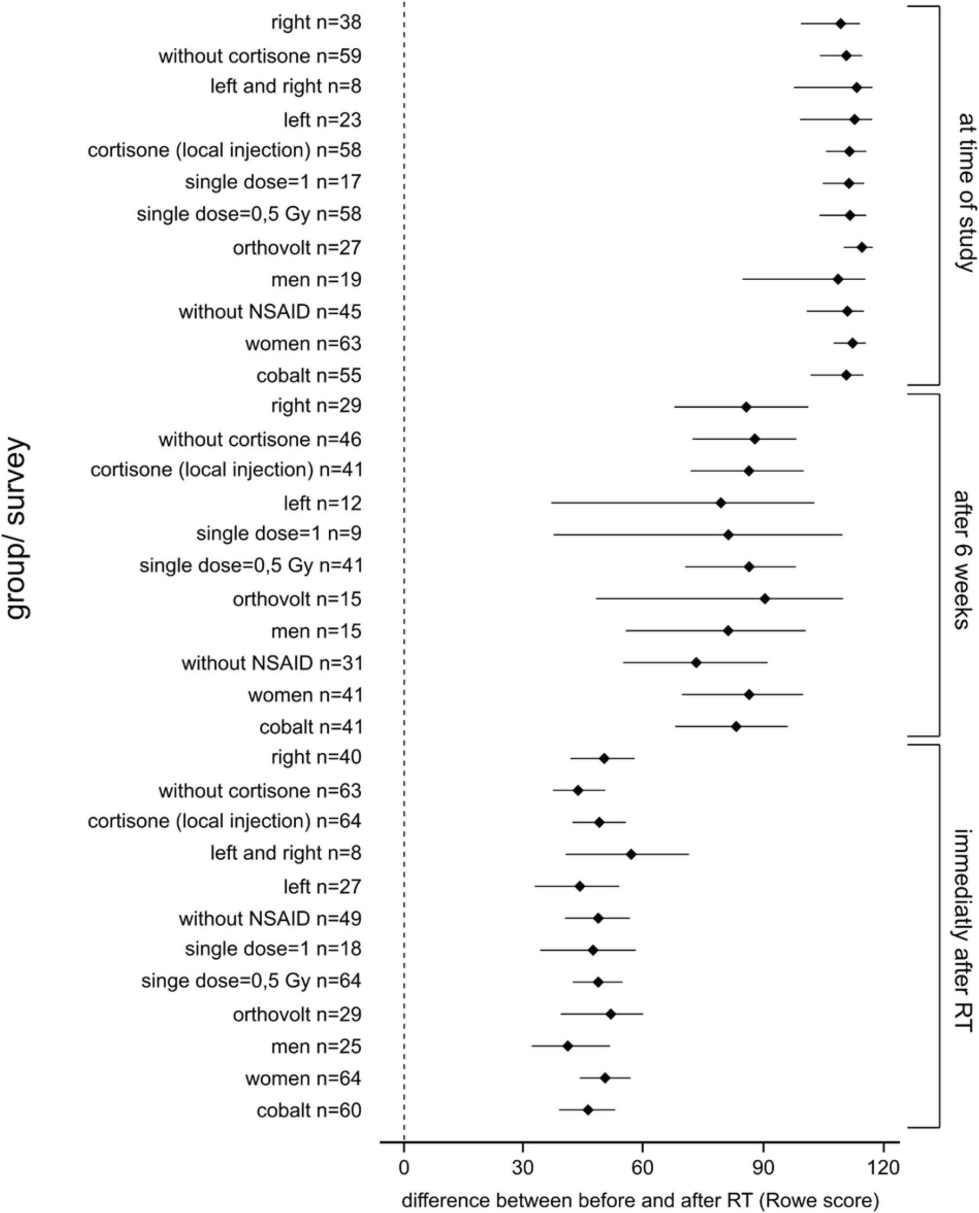
Bootstrap confidence interval of the therapeutic results for the correlation of age, total time of treatment, total dose, radiation file and the pretherapeutic time of pain to the difference of the Rowe Scores before RT, on the last day of RT, 6 weeks after RT and at the time of the final data collection. *NSAID* non-steroidal anti-inflammatory drug

**Fig. 4:**
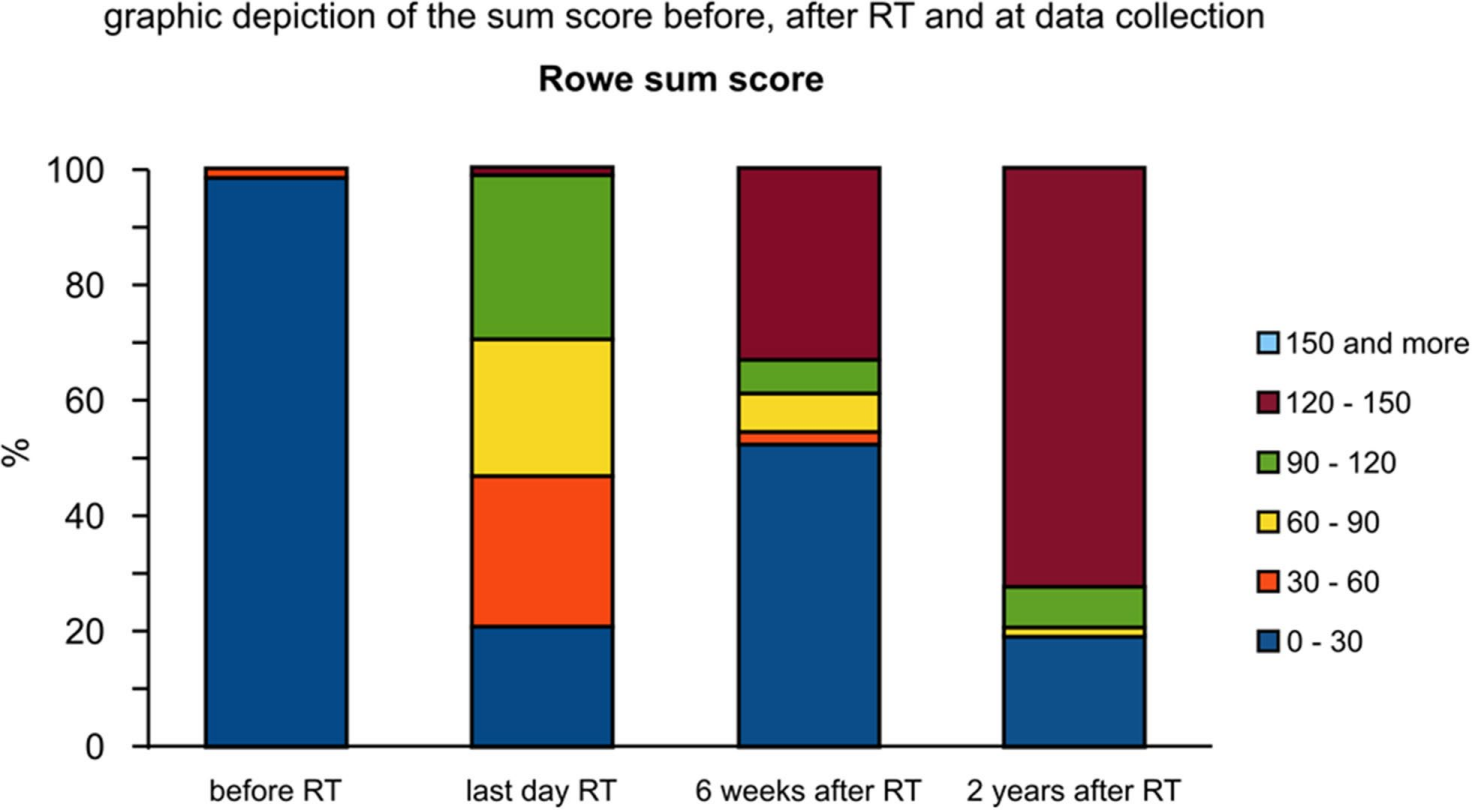
Bootstrap confidence intervals of radiation results using the modified Rowe Scores on the last day of RT, 6 weeks after RT and at data collection. Also, this investigation shows that the best results were achieved 6 weeks after RT

As presented in Figs. 3, 4 the best results were achieved 6 weeks after radiation therapy and if the radiation field was large.

The long-term response was evaluated in 102 patients after a median follow-up period of 94.4 months (range 36–187 months). 16 patients received 2 irradiation series (4 patients received orthovoltage radiotherapy, 9 patients received irradiation using cobalt and 3 patients both). Furthermore, the influence of different parameters (radiotherapeutic and clinical) on the therapeutic response (pain reduction) of the irradiation of heel spurs was evaluated. There were no significant differences between the groups receiving different single doses at any time point (*p*-value: on the last day of RT: 0.922, 6 weeks after RT: 0.865, at data collection: 0.949). Similarly, no significant differences following the Pearson correlation could be identified between the total dose (3 or 6 Gy) and the therapeutic results according to the Rowe Scores on the last day of irradiation (*r* = − 0.157, *p* = 0.172), 6 weeks after completion of radiotherapy (*r* = 0.013, *p* = 0.905) and at data collection (*r* = − 0.061, *p* = 0.670). Moreover, no significant influence of the total treatment time on any outcome was detected using the Pearson correlation: on the last day of radiotherapy (*r* = 0.06, *p* = 0.5), 6 weeks after irradiation (*r* = 0.022, *p* = 0.87) and at data collection (r = − 0.12, *p* = 0.27).

Regarding the size of the radiation fields, there was a significant influence (*p* = 0.011, *r* = − 0.0282) of the field size on the clinical outcome at 6 weeks after irradiation. This indicates a better effect of radiotherapy when an extended field was used, to irradiate the complete pain expansion on the heel. Nevertheless, this effect did not influence the early outcome on the last day of irradiation (*r* = − 0.052, *p* = 0.657) and the late outcome at data collection (*r* = − 0.036, *p* = 0.806). A pretreatment of patients with oral NSAIDs had a significant effect (*p* = 0.047) 6 weeks after completion of radiotherapy. While patients who did not receive NSAIDs (*n* = 23) had an average score of 65.22 (SD = 29.52), patients undergoing NSAID therapy (*n* = 32) achieved an average score of 51.25 (SD = 24.05). This indicates that pretreated patients who continued NSAID therapy experienced a lesser pain improvement than patients receiving radiotherapy without NSAIDs. This effect was not significant at long-term follow-up.

Though not performed via randomization, we analyzed any effect of local steroid injection on therapeutic outcome. We identified a significant lapse of pain reduction when local corticosteroid injection on the last day of radiotherapy was administered (*p* = 0.036). 31 patients without prior corticoid therapy had an average score of 34.19 (SD = 16.6) compared to 24 patients who had received corticoid injections reported an average score of 23.7 (SD = 20.6). This significant difference may indicate that patients receiving local infiltrations on the last day of radiotherapy experienced less pain reduction than patients without corticoid therapy. It could also be that these patients have a more persistent plantar enthesophyte and that there has been no improvement even after many pre-therapies. This significance was also observed 6 weeks after radiotherapy as well (*p* = 0.001), while it was not significantly different at long-term outcome (*p* ≥ 0.05).

There was no significant impact of the age of the patients on the therapy outcome on the last day of radiotherapy (*r* = 0.17, *p* = 0.12) and at long-term follow-up (*r* = 0.078, *p* = 0.49). Nevertheless, there was a significant influence of the patients’ age on the therapeutic results 6 weeks after radiotherapy (*r* = 0.28, *p* = 0.041). Higher age was significantly associated with improved outcome 6 weeks after irradiation.

Using the Mann–Whitney *U*-test, we were able to demonstrate that neither the patients’ gender, nor the technique used for radiotherapy did influence the outcome at any time. The symptomatic time before the beginning of radiotherapy showed a significant correlation on long-term outcome (*p* = 0.012, *r* = 0.315). Patients receiving irradiation a few months after the onset of plantar enthesophyte pain benefited significantly more from radiotherapy and experienced a higher pain remission than patients with delayed irradiation.

In the present study, we have categorized pain into functional scores. The pain was subdivided into pain at rest, pain at motion and pressure pain. Overall, 33 patients (32.7%) reported a complete pain regression six weeks after RT. After a follow-up of 94.4 months (range: 36–187 months), 74 patients (73%) achieved a complete response. These results confirm the effectivity of this treatment approach. The analysis of functional scores revealed that 85.5% of the patients had none or only minor restrictions in daily activities and 89% had a normal gait pattern.

## Discussion

Degenerative and inflammatory diseases of the musculoskeletal system make up 70% of all benign diseases treated by irradiation [3, 5, 6, 22, 29–40]. In Germany, more than 3500 patients annually are treated with this disease [41]. The use of low-dose irradiation when treating degenerative and inflammatory diseases has a long tradition in middle-European countries, especially in Germany [41–45].

Low-dose radiation decreases the expression and activity of glutathione peroxidase (Gpx) and nuclear factor erythroid 2-related factor 2 (Nrf2) in endothelial cells. The adhesion of peripheral blood mononuclear cells is also reduced. Large et al. provided evidence for an anti-inflammatory effect in endothelial cells stimulated by inflammation after low-dose radiation (largest effect after irradiation using 0.5 Gy) [46].

Historically, plantar enthesophytes were treated surgically in most symptomatic patients. Since surgery carries a high risk of recurrence, it is rarely performed today. Moreover, the treatment focus on symptomatic pain relief. Physiotherapy, footbed insert and medical treatment using NSAID is associated with a high therapeutic failure rate. Multiple studies in German-speaking countries reported the benefits of radiation treatment in the treatment of plantar enthesophytes in the past [6, 22, 25–27, 41, 43, 44, 47–56].

Table 3 shows the results of 19 publications between 1924 and 2004 with a total number of 3325 cases. Radiation concepts included multiple dose schemes and radiation qualities. On average, a complete pain response was achieved in 55.8% of the cases. Partial pain responses were reported in 31.2%, while 13.0% had no improvement after radiation therapy. Overall response rates varied between 65 and 100% (mean: 87.6%). We used these cases as a control group for our patients.

**Table 3:** Summary of previous published studies

Publication	Patients	Dose (Gy)	Technique	Response-rate (%)	CR (%)	PR (%)	NR (%)
	*n*	ED/GD					
Richarz (1924) [57]	5	–	Orthovolt	100	80	20	0
Pannewitz (1933) [58]	88		Orthovolt				
Mustakallio & Laitinien (1939) [59]	17	1.0–1.5/4.0–6.0	Orthovolt	82	76	6	18
Cocchi (1943) [60]	6	1.8/9.0	Orthovolt	83	33	50	17
Pizon (1957) [61]	3	–	Orthovolt	100	100	0	0
Wieland & Kuttig (1965) [62]	16	1.0/4.0	Cobalt-60	100	74	13	13
Mitrov & Harbov (1967) [63]	1520	0.5–1.5/3.0–9.0	Orthovolt	88	50	38	12
Zschache (1972) [64]	49	0.74–1.5/2.25–4.5	Orthovolt	86	12	74	14
Mantell (1978) [65]	26	2.0/10.0	240–300 kV	65	53	12	35
Basche et al. (1980) [66]	102	0.3–0.5/4.0	120 kV	90	32	58	10
Sautter-Bihl et al. (1993) [67]	15	0.5–1.0/2.5–6.0	Cobalt-60	80	60	20	20
Schäfer et al. (1995) [68]	11	0.5–1.0/2.0–4.0	Cobalt-60	72	13	59	27
Seegenschmiedt et al. (1996) [69]	72	1.0/12.0	250 kV	100	67	33	0
	98	0.3–0.5/3.0–5.0	200 kV	95	72	23	5
Lederer et al. (1998) [70]	21	1.0/6.0	4–6 MV, cobalt-60	91	43	48	9
Oehler & Hentschel (2000) [71]	258	–	Orthovolt	88	81	7	12
Koeppen et al. (2000) [72]	673	0.3/1.5–3.0	250 kV	78	13	65	22
Schreiber et al. (2000) [73]	87	1.0/6.0	6MV	86	67	19	14
Heyd et al. (2001) [74]	105		6MV	88	46	42	12
Glatzel et al. (2001) [75]	161	1.0/6.0–12.0	175 kV	89	63	26	11
Mücke et al. (2003) [76]	117	0.5/5.0–1.0	6MV	89	73	16	11
Schneider et al. (2004) [77]	68	0.25–1.0/5.0	10MV	90	53	37	10
Heyd et al. (2006) [78]	252	1.0/6.0	6MV	85.6	44.3	19.7	15.4
Heyd et al. (2007) [79]	130	0.5–1.0/3.0–6.0	6MV	87.7			12.3
Niewald et al. (2012) [80]	66	1.0–0.1/6.0–0.6	6MV				

*CR* complete remission, *ED* single dose, *GD* total dose, *NR* no remission, *PR* partial remission

Despite comprehensive clinical results, the optimal radiation dose concept needs yet to be identified. In the pattern-of-care study by Micke and Seegenschmiedt [41], single doses varied from 0.3 to 1.5 Gy and total doses from 2.5 to 18.75 Gy. In most institutions, two (44%) to three (37.5%) weekly fractions of 0.5 to 1.5 Gy were utilized. However, the authors failed to demonstrate a dose relationship. Seegenschmiedt et al. [6] identified in his study the best response rates if patients received 5 Gy or 12 Gy total dose, while patients with only 3 Gy total dose had significantly worse outcome.

Heyd et al. recently published a prospective, randomized study comparing the effect of 3 Gy (6 fractions of 0.5 Gy) vs. 6 Gy (6 fractions of 1 Gy). Both groups demonstrated a significant improvement of symptoms. A different effect of the applied radiation dose was not reported.

Our study identified size of the radiation field as a significant factor for the treatment response 6 weeks after radiation (*p* = 0.011). At the time of long-term follow-up, this effect could no longer be demonstrated. Comparable results had not been reported previously.

Previous treatments with NSAID or local steroid injections had a detrimental effect on the response to therapy. NSAID-pretreated patients had a reduced pain response at 6 weeks after RT and corticoid injections led to a worse response at the last day of RT and 6 weeks hereafter. We were unable to obtain any comparable data from the literature.

Statistical analysis provided evidence that a short pretherapeutic pain duration [6, 22, 26, 54, 81] and an increased patients’ age [6, 26, 54] were prognostic factors for improved outcome. Glatzel and coauthors reported an improved outcome when pain duration was below 12 months or the patient’s age above 50 years [26]. Similarly, Mücke [43] and Schneider [54] also reported a significantly better pain response with a pretherapeutic pain duration of < 6 months [81].

Acute and late side effects of radiation treatment have not been studied in the present population. Some authors describe an initial pain exacerbation probably due to a probably localized acidotic tissue reaction [82]. In theory, any radiation therapy may be associated with an elevated risk of cancer [83–85]. Using the low intensity as performed here, it is unlikely for developing any malignant transformation, though unmasking this possibility may take years.

This study is limited by the retrospective study design. Furthermore, the investigated cohorts were irradiated with different concepts and techniques, which strongly reduces the validity of this study.

Furthermore, in consideration of the benign condition, the length of the follow-up is not sufficient for a conclusive evaluation concerning the role of radiotherapy. Nevertheless, this study represents real-life data from a large cohort of patients with high levels of pain from a disease that has not been sufficiently investigated by large prospective trials, providing results to help inform radiotherapy decision-making.

## Conclusion

In addition to conservative treatment using physiotherapy, lifestyle changes, oral medication and orthopedic devices, radiotherapy can be regarded as a treatment option for painful plantar enthesophyte. Low radiation doses lead to a significant long-term pain reduction in more than 60% of the treated patients. In accordance with previous publications [22, 26, 43, 54, 86], we found that pretreatment pain duration was a significant prognostic factor for treatment response. In future prospective studies further associated parameters should be evaluated. In order to better assess the role of radiotherapy, prospective studies or analyses with a longer follow-up would be desirable.

## Data Availability

All data and materials can be accessed via CM and FD.

## Abbreviations

Co: Cobalt
Gy: Gray
NSAID: Non-steroidal anti-inflammatory drugs
RT: Radiotherapy
SD: Standard deviation
S-Score: Sum-Score
